# Clinicopathological and Radiological Correlation Among the Spectrum of Nodular Thyroid Lesions

**DOI:** 10.7759/cureus.70725

**Published:** 2024-10-02

**Authors:** Keerthana Rajyakodi, Archana Balasubramanian, Sandhya Sundaram, Harini Gnanavel

**Affiliations:** 1 Pathology, Sri Ramachandra Institute of Higher Education and Research, Chennai, IND; 2 Radiology, Sri Ramachandra Institute of Higher Education and Research, Chennai, IND

**Keywords:** discordance, follicular neoplasm, niftp, nodular lesion, papillary carcinoma, risk of malignancy, tirads

## Abstract

Objective

The primary objective of this study is to comprehensively evaluate nodular thyroid lesions. By analyzing the morphological characteristics of surgically resected specimens and biopsies, the aim is to establish correlations between the patterns, corresponding clinical diagnoses and radiological findings. Furthermore, the study will investigate cases where there is a discrepancy between imaging and pathological assessments, with the goal of understanding the contributing factors to this discordance and improving diagnostic accuracy.

Methodology

This retrospective observational study utilized convenience sampling to recruit participants. Sample size was determined using GPower software (Heinrich-Heine-Universität Düsseldorf, Düsseldorf, Germany). Statistical analysis was performed using Jamovi 2.3.28 (Jamovi Research, Vienna, Austria). The Chi-square test was employed to assess the association between the Thyroid Imaging Reporting and Data System (TIRADS) score and histopathological diagnosis. Receiver operating characteristic (ROC) analysis was conducted to identify the optimal TIRADS cut-off point for classifying nodules as malignant and to evaluate the diagnostic accuracy of TIRADS. Discordant cases between histopathological and radiological findings were analyzed to investigate potential discrepancies.

Result

Taking into consideration histopathology report as a gold standard, correlation of TIRADS with histopathology findings, TIRADS showed 73.08% sensitivity and 81.08% specificity, showing the strongest balance between sensitivity and specificity, as demonstrated by the Youden's Index and Metric Score. The area under the curve (AUC) remains constant at 0.805, suggesting a consistent overall discriminative ability of the TIRADS scale.

Conclusion

A primary advantage of this study is its exclusive focus on surgically resected nodules, which allows for definitive histological confirmation and thereby ensures the most accurate diagnostic assessment.

## Introduction

Thyroid nodules are a common clinical occurrence, with a reported prevalence of approximately 8.5% in the general population [1]. Among the sexes, women exhibit a higher incidence when compared to men, especially among elderly age groups [2,3].

Ultrasound is the predominant imaging modality employed for initial thyroid nodule assessment. The American College of Radiology Thyroid Imaging, Reporting and Data System (ACR TIRADS) was implemented in 2017 as a standardized framework for ultrasound-based risk assessment of thyroid nodules [4]. It employs a five-tiered classification (TIRADS 1-5) based on a scoring system derived from specific ultrasound features: composition, echogenicity, shape, margins, and the presence of echogenic foci. Higher TIRADS categories correspond to increased malignancy risk. Subsequent management decisions, including fine-needle aspiration biopsy, follow-up imaging, or observation, are guided by the category of TIRADS nodule and size.

However, the limitations of ultrasound in differentiating benign from malignant nodules are well-established due to overlapping sonographic features. Consequently, definitive diagnosis typically requires histopathological examination of surgically resected tissue. This study aims to evaluate nodular thyroid lesions, correlate their morphology with clinical and radiological findings, and investigate imaging-pathology discordance.

## Materials and methods

Ethical considerations and study setting

The present study was conducted over two years. This retrospective observational study was done at a tertiary care hospital and was approved by the Institutional Ethics Committee (CSP-MED/24/JUL/106/234), Sri Ramachandra Institute of Higher Education and Research, prior to the study.

A convenience sampling technique was used to recruit the participants. All participants were interviewed with a questionnaire, which included the demographic characteristics, symptom assessment, and clinical diagnosis. The histopathology and radiological findings were obtained from the Departments of Pathology and Radiology, respectively.

Sample size and sampling technique

Based on the previous study by Gayathri et al., the agreement between the histopathological and radiological findings for patients with thyroid nodules was 0.84%. With a cue from the above study, at 5% level of significance, 80% power, and to defect 10% shift in the agreement between the radiology and histological findings, the estimated sample size was 79. The sample size was estimated using GPower software version 3.1 (Heinrich-Heine-Universität Düsseldorf, Düsseldorf, Germany). With a 15% attrition rate, the required sample size was 92 [5].

Study procedure

The Thyroid Imaging Reporting and Data System (TIRADS) was used to categorize thyroid nodules based on ultrasound features. The patient lying on their back with their neck extended, a high-frequency transducer was used to obtain detailed images of the thyroid gland. The nodules were assessed for composition, echogenicity, margins, calcifications, and shape. Based on the above assessments, the nodules were classified into TIRADS 1 (benign), TIRADS 2 (not suspicious), TIRADS 3 (mildly Suspicious), TIRADS 4 (moderately suspicious), and TIRADS 5 (highly suspicious); larger the TIRADS score, higher the risk of malignancy.

A total of 100 cases of partial or complete thyroidectomy were studied. The tissues were processed with paraffin block, and slides were created with was then sectioned with sizes ranging from 3 to 5 µm. Hematoxylin and eosin (H&E) staining was then performed. Microscopic examination was then performed by a pathologist. Based on the microscopic findings, the final histopathology diagnosis was arrived as either benign or malignant.

Study participants

This study included all patients who presented with thyroid swellings and underwent surgical treatment, regardless of age. To ensure a homogeneous group with comprehensive diagnostic information, patients were excluded if either histopathological analysis or imaging findings were not available. Histopathological analysis was based on surgical resection specimens, excluding patients who only underwent fine-needle aspiration (FNA) biopsies. Imaging criteria included specifically ultrasound with Thyroid Imaging Reporting and Data System (TIRADS) classification; patients with only computed tomography (CT) or ultrasonography (US) without TIRADS were also excluded. This approach allowed for a more focused analysis based on patients with complete diagnostic data, including a standardized ultrasound classification system (TIRADS) for nodule characterization.

Statistical considerations

The data entry was performed in Microsoft Excel version 2019 (Microsoft Corp., Redmond, WA); double data entry method was performed to prevent any potential for data mismatches and missing data. The data is then imported into Jamovi version 2.3.28 (Jamovi Research, Vienna, Austria) for statistical analysis. Categorical variables are expressed as percentage, and for association between the TIRADS score and histopathological diagnosis, Chi-square (χ²) test was applied. TIRADS score was considered as both categorical (risk grade) and continuous scale during the analysis. To test the optimal cut-off point of the TIRADS to classify the nodules as malignant and to calculate the diagnostic accuracy of TIRADS, receiver operating characteristic (ROC) analysis was performed. All tests were 2-tailed, and a p value of less than 0.05 was considered significant. The study involved a comparison of histopathology and radiology findings, identifying instances of discordance that were subsequently analyzed in details.

## Results

A total of 100 patients were included in this study. The highest incidences of thyroid enlargement were found in 88 females (88%), with males comprising 12 (12%), in the ratio of 7:1. The most common clinical symptom in the patients with thyroid lesions was swelling in the neck which was present in all cases. Multinodular goiter was the most common radiological finding seen in 83 cases, solitary nodular goiter was seen in 14 cases, and three cases were diffuse goiter.

Out of 100 cases, 74 (74%) cases were benign and 26 (26%) cases were malignant. Out of 12 male patients, five cases (41.6%) were malignant. The most common histopathological diagnosis was adenomatoid nodule, which accounts for 41 (41%) cases. Papillary carcinoma thyroid is the next most frequent at 23 (23%) among the 23 cases, three were microcarcinoma (classic) <1 cm. Colloid nodules and lymphocytic thyroiditis were equally prevalent, representing 12 (12%) cases. Other diagnoses such as benign thyroid cyst, non-invasive follicular thyroid neoplasm with papillary-like nuclear features (NIFTPs), follicular adenoma, oncocytic adenoma, follicular carcinoma, and medullary carcinoma are less common. The distribution of patient characteristics, clinical diagnosis, histology, and histopathological diagnosis in the study population is shown in Table 1.

**Table 1:** Distribution of Patient Characteristics, Clinical Diagnosis, Histology, and Histopathological Diagnosis in the Study Population Data presented as frequency (n) and percentage (%) of all the cases. NIFTP: non-invasive follicular thyroid neoplasm with papillary-like nuclear features.

		Frequency (n=100)	Percentage (%)
Sex	Female	88	88.0%
	Male	12	12.0%
Clinical diagnosis	Multinodular	83	83.0%
	Solitary nodular goiter	14	14.0%
	Diffuse enlargement	3	3.0%
Histology	Adenomatoid nodule	41	41.0%
	Benign thyroid cyst	2	2.0%
	NIFTP	2	2.0%
	Colloid Nodule	12	12.0%
	Papillary carcinoma thyroid	23	23%
	Medullary carcinoma thyroid	1	1%
	Lymphocytic thyroiditis	12	12.0%
	Follicular adenoma	4	4.0%
	Oncocytic adenoma	1	1.0%
	Follicular carcinoma	2	2.0%
Histopathological Diagnosis	Benign	74	74.0%
	Malignant	26	26.0%

Table 2 presents the frequency distribution of patients across different TIRADS (Thyroid Imaging Reporting and Data System) categories. The majority of cases fall into TIRADS 3, which accounts for 40 cases (40%). TIRADS 2 was the second most common category, comprising 25 cases (25%). TIRADS 4 and 5 categories, which are associated with a higher risk of malignancy, represent 23 (23%) and 10 (10%) cases, respectively.

**Table 2:** Distribution of Patients According to TIRADS Classification Data presented as frequency (n) and percentage (%). TIRADS: Thyroid Imaging Reporting and Data System.

TIRADS	Frequency (n=100)	Percentage (%)
1	2	2
2	25	25
3	40	40
4	23	23
5	10	10

**Figure 1:**
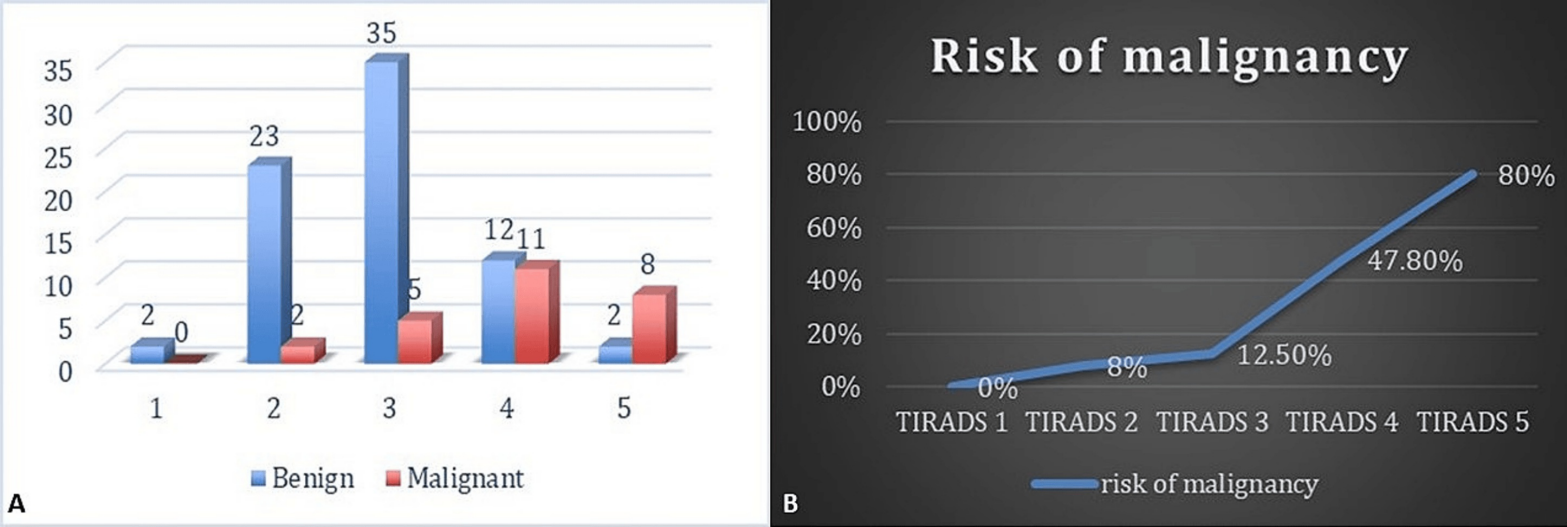
Distribution of Benign and Malignant Cases and Risk of Malignancy Across Different TIRADS Categories. A. The histogram illustrating the distribution of benign and malignant cases across different TIRADS categories. The blue bars represent the number of benign cases, while the red bars represent the number of malignant cases in each TIRADS category. B. The line graph illustrates the increasing risk of malignancy across different TIRADS categories. TIRADS: Thyroid Imaging Reporting and Data System.

The TIRADS categories exhibit varying risks of malignancy. TIRADS 1, which includes only two cases, shows no malignant outcomes, indicating it as a low-risk category. TIRADS 2 predominantly consists of 23 (31.1%) benign cases with a small proportion of malignancies two (7.7%) cases, suggesting a generally low risk of malignancy. TIRADS 3 demonstrates five malignant cases (19.2%) and 35 benign cases (47.2%). In contrast, TIRADS 4 presents a significant proportion of malignancies 11 cases (42.3%) and few benign cases 12 (16.2%), reflecting a higher risk of malignancy. TIRADS 5 also shows a high percentage of eight malignant cases (30.8%) and a very low percentage of benign cases, marking it as a high-risk category (Table 3). Overall, the data underscores that higher TIRADS categories (4 and 5) are more strongly associated with malignant outcomes, while lower TIRADS categories (1 and 2) tend to be more benign (Figure 1A).

**Table 3:** Cross-tabulation of TIRADS Categories with Benign and Malignant Outcomes TIRADS ranging from 1 to 5, with varying counts and percentages for each diagnosis. It highlights the frequency and percentage of each histological diagnosis within each TIRADS category and the overall distribution across all categories. TIRADS: Thyroid Imaging Reporting and Data System.

TIRADS	Benign	Malignant	Total	P value
1	2	0	2	<0.001
2	23	2	25	
3	35	5	40	
4	12	11	23	
5	2	8	10	
Total	74	26	100	

The distribution of histological diagnoses across different TIRADS categories is shown in Table 4. The majority of cases, 40 (40%), fall into TIRADS 3, with 25 cases (61%) of adenomatoid nodules, five cases (41.6%) of lymphocytic thyroiditis, and four cases (17.4%) of papillary carcinomas classified in this category. TIRADS 2 includes 25 (25%) cases, most notably seven cases (58.3%) of colloid nodules and five cases (41.7%) of lymphocytic thyroiditis. TIRADS 4 accounts for 23 (23%) cases, with 10 (43.5%) papillary carcinomas. TIRADS 5 comprises 10 cases (10%), predominantly featuring seven papillary carcinomas (30.4%). TIRADS 1 includes only two (2%) cases, with a small representation of adenomatoid nodules and lymphocytic thyroiditis.

**Table 4:** Distribution of TIRADS Categories with Histological Diagnoses NIFTP: non-invasive follicular thyroid neoplasm with papillary-like nuclear features, TRIADS: Thyroid Imaging Reporting and Data System.

TIRADS	Adenomatoid Nodule	Benign Cyst	Colloid Nodule	Follicular Adenoma	Follicular Carcinoma	Oncocytic Adenoma	Lymphocytic Thyroiditis	NIFTP	Papillary Carcinoma	Medullary carcinoma	Total	% within Histology
1	1	0	0	0	0	0	1	0	0	0	2	2%
2	9	1	7	0	0	0	5	1	2	0	25	25%
3	25	1	2	2	0	0	5	0	4	1	40	40%
4	5	0	2	2	1	1	1	1	10	0	23	23%
5	1	0	1	0	1	0	0	0	7	0	10	10
Total	41	2	12	4	2	1	12	2	23	1	100	1

The line graph illustrates the increasing risk of malignancy across different TIRADS categories. At TIRADS 1, the risk is 0%, indicating no malignancy. This risk slightly increases to 8% at TIRADS 2 and continues to rise to 12.5% at TIRADS 3. A significant jump is observed at TIRADS 4, where the risk of malignancy reaches 47.8%. The highest risk is seen at TIRADS 5, with an 80% probability of malignancy. The graph shows a clear upward trend, reflecting the escalating risk of malignancy as the TIRADS category increases (Figure 1B).

TIRADS 1 on the TIRADS scale exhibits 100% sensitivity but 0% specificity, meaning it correctly identifies all malignant cases but cannot distinguish benign ones, resulting in a high rate of false positives. The Youden's Index and Metric Score are the lowest, and the negative predictive value (NPV) is not calculable.

TIRADS 2 also maintains 100% sensitivity with a slight improvement in specificity to 2.7%, but the specificity remains low, with only a minimal increase in diagnostic accuracy. TIRADS 3 offers a more balanced performance, with a sensitivity of 92.31% and specificity of 33.78%, indicating a better balance between true positives and false positives, as reflected in higher Youden's Index and Metric Score. TIRADS 4 provides an optimal trade-off with 73.08% sensitivity and 81.08% specificity, showing the strongest balance between sensitivity and specificity, as demonstrated by the highest Youden's Index and Metric Score. TIRADS 5 achieves the highest specificity at 97.3% but has the lowest sensitivity at 30.77%, indicating that while it is highly specific in identifying benign cases, it misses a significant number of malignant cases. Despite the variation in sensitivity and specificity across these TIRADS, the area under the curve (AUC) remains constant at 0.805, suggesting a consistent overall discriminative ability of the TIRADS scale. This analysis helps determine that TIRADS 4 might offer the best balance between sensitivity and specificity for clinical use (Table 5).

**Table 5:** Diagnostic Performance of TIRADS. TIRADS: Thyroid Imaging, Reporting and Data System, PPV: positive predictive value, NPV: negative predictive value, AUC: area under the curve, #N/A: Not applicable or no value is available. This table illustrates sensitivity (%), specificity (%), PPV (%), NPV (%), Youden's Index, AUC, and Metric Score.

Cutpoint	Sensitivity (%)	Specificity (%)	PPV (%)	NPV (%)	Youden's Index	AUC	Metric Score
1	100%	0%	26%	#N/A	0.0000	0.805	1.00
2	100%	2.7%	26.53%	100%	0.0270	0.805	1.03
3	92.31%	33.78%	32.88%	92.59%	0.2609	0.805	1.26
4	73.08%	81.08%	57.58%	89.55%	0.5416	0.805	1.54
5	30.77%	97.3%	80%	80%	0.2807	0.805	1.28

**Figure 2:**
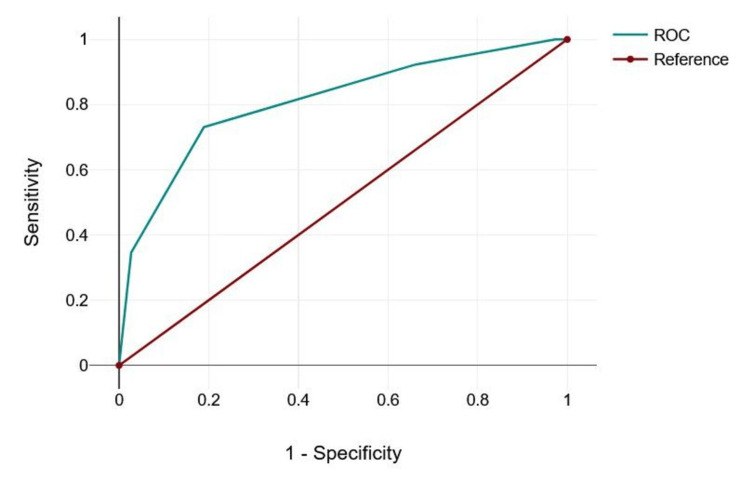
ROC Curve Analysis of TIRADS. ROC: receiver operating characteristic, TIRADS: Thyroid Imaging Reporting and Data System.

Figure 2 shows receiver operating characteristic (ROC) curve for TIRADS in predicting the thyroid nodule malignancy compared with histopathological classification of the nodule. The area under the curve was 0.805, indicating excellent test.

Three cases of discordance between imaging and pathology results were identified, offering significant learning opportunities.

Discordant case 1

**Figure 3:**
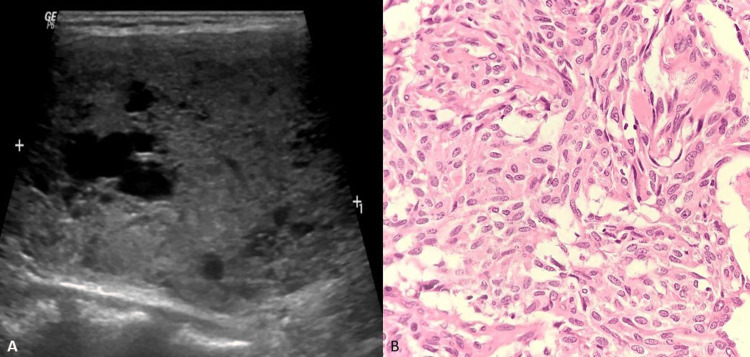
USG and Hematoxylin and Eosin Stain to Discordant Case 1. A. USG of left thyroid show smooth, well-defined, mixed echogenic, solid-cystic nodule categorized as TIRADS 3. B. Hematoxylin and eosin stain of medullary carcinoma thyroid (×400 magnification). The tumor cells are spindle-shaped cells arranged in the nest with eosinophilic granular cytoplasm with stippled chromatin. USG: ultrasonography, TIRADS: Thyroid Imaging Reporting and Data System.

A 50-year old female presented with a swelling in front of her neck and underwent ultrasonography, which showed a TIRADS 3 smooth, well-defined, mixed echogenic, solid-cystic nodule of size 3.5x2.7 x 5.4 cm, replacing entire left lobe of thyroid (Figure 3A). Histopathology revealed a diagnosis of medullary carcinoma thyroid (Figure 3B). The reason for discordance is an unusual finding for medullary carcinoma. Preoperative ultrasonography typically demonstrates nonspecific malignant characteristics including hypoechoic, solid masses with irregular borders, and internal vascularity. The hypoechoic nodule with calcifications and irregular borders was classified at ultrasonography as suspicious.

Discordant case 2

**Figure 4:**
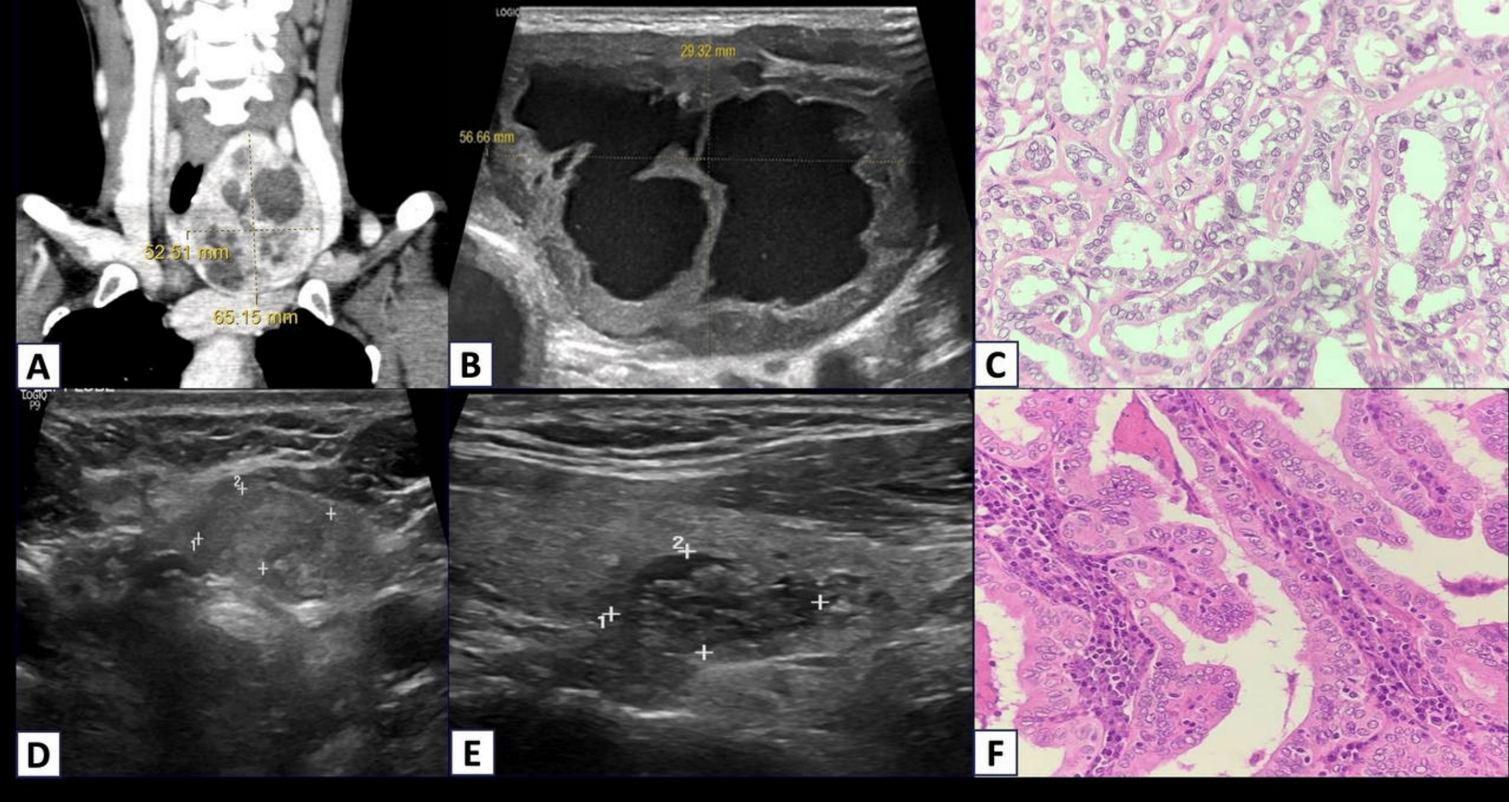
CT and USG to Discordant Cases 2 and 3. Case 2 (A-C): A. CT neck plain and contrast. B. USG thyroid show TIRADS 3 cystic lesion replacing the entire thyroid TIRADS 3. C. Histology of follicular variant of papillary carcinoma thyroid (H&E stain ×200 magnification), showing tumor cells arranged in follicular architecture with nuclear features of papillary thyroid carcinoma. Case 3 (D-F):  D and E. USG thyroid show smooth, well-defined, mixed echogenic nodules are wider than tall, with no calcification (TIRADS 2). F. H&E papillary thyroid carcinoma ×200 magnification, showing that tumor cells are arranged in papillary architecture with nuclear features showing crowding, grooving, and nuclear enlargement with clearing of nuclei. CT: computer tomography, USG: ultrasonography, H&E: hematoxylin and eosin, TIRADS: Thyroid Imaging Reporting and Data System.

A 49-year-old woman developed a gradually growing neck swelling over six months. CT Imaging studies revealed a large, well-defined, solid-cystic lesion of size 7.5 x 5.8 x 4.5 cm in the left thyroid lobe and isthmus with no obvious extracapsular extension, favoring benign etiology (Figure 4A). Preoperative ultrasound also showed predominantly cystic lesion replacing the entire thyroid (TIRADS 3) (Figure 4B). Despite initial indications of a benign nature, histopathology confirmed a diagnosis of follicular variant papillary thyroid carcinoma (FVPTC) (Figure 4C). The common ultrasound presentation of FVPTC is solid nodule without calcification, leading to a clinico-radio-pathological discordance in this case. In one study by Mileva et al. with 112 malignant thyroid nodules, 34 (30.3%) were follicular variants of papillary carcinoma, frequently exhibiting benign ultrasonographic features [6]. Cystic components may be present within the nodule [7].

Discordant case 3

A 50-year-old woman presented with swelling in the neck. USG revealed multinodular goiter with smooth, well-defined, mixed echogenic nodules are wider than taller, with no calcification (Figures 4D, 4E). Histopathology revealed papillary thyroid carcinoma, classic subtype in both lobes (Figure 4F). The reason for the discordance in this case was the unusual radiological presentation. The typical ultrasonographic features of papillary thyroid carcinoma (PTC) include a solid, hypoechoic nodule with microcalcifications or echogenic foci and ill-defined margins [7].

## Discussion

According to the GLOBOCAN 2020 database published by the World Health Organization's International Agency for Research on Cancer, thyroid cancer ranks as the ninth most commonly diagnosed malignancy globally with about 43,720 affected adults in the United States alone [3]. A recent study in India reports a higher prevalence of palpable thyroid nodules in the community, reaching approximately 12.2% [1]. Upon subdividing the data, age-standardized incidence rates among the sexes were found to be about three times higher in women, with incidence rates of 10.1 per 100,000 women and 3.1 per 100,000 men [2,3]. Our study aligns with these epidemiological trends, demonstrating a significant female predominance among patients.

Thyroid neoplasms constitute the most prevalent endocrine malignancies. Most of these tumors originate from the follicular epithelial cells, which comprise the glandular parenchyma. Thyroid neoplasms of follicular cell origin exhibit a wide spectrum of biological behavior, ranging from benign adenomas to highly aggressive malignancies. This spectrum encompasses low-grade variants and incidental microscopic foci. As per Cancer Genome Atlas Research Network (2014), benign thyroid neoplasms, classified as adenomas, typically exhibit a micro- or macrofollicular architecture and often harbor a rat sarcoma virus (RAS)-like molecular profile [8].

The incidence of thyroid cancer has tripled over the past three decades, with concomitant shifts in histological and genetic profiles [9]. All other thyroid cancers originate from the follicular epithelial cells except for medullary carcinoma. Multinodular goiter (MNG) demonstrates a higher prevalence in women compared to men, with a female-to-male ratio ranging from 6:1 to 15:1 [10]. This study also shows similar findings.

Thyroid cells with abundant eosinophilic cytoplasm have historically been termed Hürthle cells, a misnomer as Hürthle originally described C cells. To rectify this, the term "oncocyte" is proposed. Some adenomas are composed of oncocytic cells [11]. Thyroid follicular nodular disease (TFND) is a common condition characterized by the presence of multiple benign nodules representing a spectrum from hyperplasia to neoplasia. Histological differentiation between hyperplastic and neoplastic follicular cell lesions within these nodules can be challenging. TFND may manifest clinically as multinodular goiter, although it often occurs in glands of normal size [10].

A category of low-risk neoplasms has been established to bridge the gap between benign and malignant thyroid tumors. Despite a minimal reported incidence of metastasis, these neoplasms possess a low but existent metastatic potential. To avoid overtreatment, the designation "tumor" has been adopted over "carcinoma." This category comprises neoplasms exhibiting cytological atypia without invasive features (NIFTP), those with uncertain malignant potential (UMP) [12], and those displaying a unique combination of papillary carcinoma-like nuclear characteristics and hyaline cytoplasm (hyalinizing trabecular tumors) [13].

Fukushima et al. investigated the prognostic implications of ultrasonographic features in patients with non-hereditary medullary thyroid carcinoma [14]. The study demonstrated a favorable prognosis for patients exhibiting ultrasonographic characteristics resembling those of follicular tumors or benign nodules. Their study cohort, spanning 19 years (1988-2007), comprised 77 patients classified into "malignant" (M-type, 70%) and "benign" (B-type, 30%) groups according to ultrasound appearance. B-type MTCs demonstrated an indolent clinical course with favorable outcomes and significant post-surgical calcitonin reduction. In contrast, M-type MTCs exhibited aggressive features, including lymph node involvement, extrathyroidal invasion, and persistent disease [14,15].

A systematic review by Mistry et al. (2020) on the ultrasound classification of thyroid nodules found that the sensitivity, specificity, positive predictive value, and negative predictive value of these classifications ranged from 70.6% to 97.4%, 29.3% to 90.4%, 23.3% to 64.3%, and 87.1% to 99.0%, respectively [16]. In contrast, larger studies evaluating TIRADS, such as the combined cohort of Zhang et al. (2015) and Yoon et al. (2014) encompassing 5,273 nodules from 4,162 participants, demonstrated significantly higher sensitivities ranging from 97.0% to 97.4% [17,18]. These findings suggest that the diagnostic accuracy of TIRADS improves when applied to larger datasets. The median sensitivity for TIRADS in these studies was 90.0%, with a substantial proportion exceeding this threshold, indicating its effectiveness in identifying thyroid nodules with malignant potential.

A study by Singaporewalla et al. done on a cohort of 100 participants reported a sensitivity of 70.6% for TIRADS [19]. In contrast to the relatively consistent sensitivity, TIRADS specificity exhibited significant heterogeneity across studies. While a large study by Zhang et al. (2015) demonstrated a specificity of 90.0% [17], other studies, including one by Yoon et al. (2014), reported specificities below 50% [18]. The median specificity was notably lower at 57.5%, as reported by Gharib (2016) [20]. These findings suggest that the diagnostic accuracy of TIRADS in terms of specificity may vary depending on factors such as study population and methodology.

The median positive predictive value (PPV) for TIRADS was similarly suboptimal at 49.0%. Large-scale studies involving a combined cohort of 5,273 nodules from 4,162 participants demonstrated PPVs ranging from 23.3% to 40.0% [16-18]. In this study, there is a slight improvement in PPV is 55.58%. The negative predictive value (NPV) of TIRADS demonstrated the most consistent performance across studies, with a median of 91.0% seen in the study by Gharib (2016) [20]. Notably, three of the five studies reported NPVs exceeding 90%, including two large-scale investigations with a combined cohort of 5,273 nodules that yielded NPVs between 91.1% and 98.1% [16-19]. This study also revealed similar results with a specificity of 89.55%.

Studies by Moifo et al. and Sanchez reported a lower risk of malignancy in TIRADS category 2 (0%) compared to the present study [21,22]. However, a study conducted in the Indian subcontinent by Chandramohan et al. assessed the practical aspects and accuracy of TIRADS in daily clinical practice and observed a similar predictive value for malignancy in TIRADS 2 (6.6%) as found in this study [23]. The current study identified a slightly higher risk of malignancy in TIRADS 2, at 8%.

The risk of malignancy in TIRADS 3 nodules in this study was 12.5%, consistent with the 5%-10% risk reported by Horvath et al. [24]. However, this rate was slightly higher than the 2.2% risk observed in studies by Moifo et al. and Sanchez [21,22]. In contrast, studies conducted by Chandramohan et al. and Srinivas et al. found higher rates of malignancy in TIRADS 3 nodules, at 32% and 6.4%, respectively [23,25].

The risk of malignancy in the TIRADS 4 category in this study was 47.8%, similar to the 48% reported by Sanchez [22]. While Moifo et al. observed a slightly higher risk of malignancy in TIRADS 4 (57.9%) [21], studies conducted by Chandramohan et al. and Srinivas et al. found substantially higher rates of malignancy in TIRADS 4, reporting 64% and 66.6%, respectively [23,25]. The overall risk associated with TIRADS 5 was consistent across all studies [21-25].

Although TIRADS 2 and 3 categories typically indicate a low risk of malignancy, young female patients may exhibit a higher incidence of thyroid cancer. Consequently, vigilant follow-up and evaluation are imperative in this demographic. While false negatives can occur, persistent clinical suspicion warrants further investigation. Conversely, TIRADS 5 nodules demonstrate a strong association with malignancy.

A significant advantage of this study is its exclusive focus on surgically resected nodules, allowing for definitive histological confirmation. This approach ensured the most accurate diagnostic assessment. However, the study has limitations. Its retrospective, single-center design and smaller sample size, primarily due to thyroidectomies performed for cosmetic purposes in patients with benign fine-needle aspiration cytology results, may affect the applicability of our findings to a broader population.

## Conclusions

The collaborative evaluation through interdepartmental or multidisciplinary meetings significantly improved correlation rates in complex thyroid cases. This underscores the importance of a team-based approach for accurate diagnosis. It is essential to document discordant findings in pathology reports to mitigate the risk of overlooking potential malignancies. This proactive measure helps prevent diagnostic delays and confusion, ensuring timely and appropriate treatment. Moreover, recommending follow-up in pathology reports can contribute to effective patient management by guiding ongoing monitoring.
